# Single Copies of the 5S rRNA Inserted into 45S rDNA Intergenic Spacers in the Genomes of Nototheniidae (Perciformes, Actinopterygii)

**DOI:** 10.3390/ijms24087376

**Published:** 2023-04-17

**Authors:** Alexander Dyomin, Svetlana Galkina, Arina Ilina, Elena Gaginskaya

**Affiliations:** Biological Faculty, St. Petersburg State University, Universitetskaya Emb. 7/9, St. Petersburg 199034, Russia; a.demin@spbu.ru (A.D.); svetlana.galkina@spbu.ru (S.G.); st057025@student.spbu.ru (A.I.)

**Keywords:** toothfish, ribosomal RNA, rDNA amplification, amplified nucleoli, maternal rRNA

## Abstract

In the vast majority of Animalia genomes, the 5S rRNA gene repeats are located on chromosomes outside of the 45S rDNA arrays of the nucleolar organiser (NOR). We analysed the genomic databases available and found that a 5S rDNA sequence is inserted into the intergenic spacer (IGS) between the 45S rDNA repeats in ten species of the family Nototheniidae (Perciformes, Actinopterigii). We call this sequence the NOR-5S rRNA gene. Along with Testudines and Crocodilia, this is the second case of a close association between four rRNA genes within one repetitive unit in deuterostomes. In both cases, NOR-5S is oriented opposite the 45S rDNA. None of the three nucleotide substitutions compared to the canonical 5S rRNA gene influenced the 5S rRNA secondary structure. In transcriptomes of the Patagonian toothfish, we only found NOR-5S rRNA reads in ovaries and early embryos, but not in testis or somatic tissues of adults. Thus, we consider the NOR-5S gene to be a maternal-type 5S rRNA template. The colocalization of the 5S and 45S ribosomal genes appears to be essential for the equimolar production of all four rRNAs in the species that show rDNA amplification during oogenesis. Most likely, the integration of 5S and NOR rRNA genes occurred prior to Nototheniidae lineage diversification.

## 1. Introduction

The ribosomal genes (rDNA) encoding 18S, 5.8S, 28S, and 5S ribosomal RNA (rRNA) are key elements of eukaryotic genomes, since their products are directly involved in the biogenesis and functioning of the ribosomes providing for protein synthesis. Multiple 45S rDNA clusters of the 18S, 5.8S, and 28S rRNA genes separated by intergenic spacers (IGS) form the nucleolus organiser region (NOR) that can be situated in one chromosome pair or in a small number thereof. In the vast majority of Animalia genomes, copies of the 5S rRNA gene are not linked to NORs; they can be grouped at one or several chromosome sites outside the NOR as reviewed in [1,2,3,4]. The existence of this pattern correlates with different pathways of 45S and 5S rRNA biogenesis. The 18S, 5.8S, and 28S rRNA genes are transcribed by RNA polymerase (RNAP) I to a single precursor and then processed into three separate rRNA molecules in the nucleolus. The 5S rRNA molecules, synthesised by RNAP III, move into the cytoplasm through nuclear pores to complete processing. After that, they are imported back to the nucleus where they reach the nucleolus to be incorporated into the large ribosomal subunit [5]. In the genome of an organism, the 45S and 5S ribosomal genes are usually represented by different numbers of copies. For example, in humans, the diploid copy number varies from about 60 to more than 800 units for 45S rDNA [6,7,8,9,10], and from about 10 to more than 400 copies for 5S rDNA [9]. The existence of separate 45S and 5S rDNA arrays transcribed by specialised polymerases suggests their concerted transcription since all rRNA molecules are present in the ribosome in equimolar amounts (in a single copy). A certain balance of rRNA molecules in a cell can be maintained due to the fact that not all rDNA repeats in the NOR are active at the same time [11,12].

Impressive mechanisms that maintain the balance between the 45S pre-rRNA and 5S rRNA genes have been found in animals with 45S rDNA amplification in oocytes. In vertebrates, multiple NOR rDNA amplification accompanied by formation of numerous extrachromosomal nucleoli was described for oocytes of fish [13,14,15], amphibians [16,17,18,19,20], turtles [21,22,23], and crocodiles [24,25]. In these cases, the apparent imbalance between the doses of 18S, 5.8S, 28S, and 5S rRNA genes was counterbalanced by the presence of additional copies of 5S rRNA genes transcribed differentially in somatic cells and oocytes. In the genomes of some amphibians and fish, two types of slightly different 5S rRNA genes have been described [15,18,26,27,28]; their copies forming separate arrays outside the NOR. These two types of 5S rDNA, the oocytic (or maternal) and the somatic, show a differential pattern of gene expression in different tissues. In *Xenopus* species, the somatic 5S rDNA type is transcribed in the course of oogenesis, late embryogenesis, and in adult tissues [29]. It occupies a single locus on chromosome 9 in both *X. laevis* and *X. borealis* [30]. The other variant (the oocytic type), located at the distal end of the long arms of most *Xenopus* chromosomes [17,30], is transcribed in the growing oocytes and is inactive in early embryos and in the somatic cells of adults [30,31,32]. This genomic organisation is consistent with that shown in zebrafish [15]. In *Danio rerio*, the maternal type 5S rDNA contains several thousands of gene copies and is located on chromosome 4, whereas the somatic type set of only 12 gene copies is situated on chromosome 18. The oocytic and somatic 5S rRNA gene locations and expressions have only been thoroughly studied in the teleost species mentioned above; however, they were also found in *Tinca tinca* [33] and *Misgurnus fossilis* [34], and described in the genomes of other fish reviewed in [35], such as the representatives of the genus *Leporinus* [36] and *Oreochromis niloticus* [37]. This suggests a similar pattern of 5S rDNA organisation, with the oocytic and somatic 5S rDNA arrays occupying separate chromosome loci.

We have recently shown that in the genomes of turtles (Testudines) and crocodiles (Crocodilia), the oocytic/somatic 5S rDNA is organised differently. The oocyte 5S rRNA gene is inserted into the IGS of the NOR rDNA repeats [38]. This NOR-5S rRNA gene differs by 20% from the canonical 5S rRNA gene, referred to as the NTS-5S rRNA gene. The internal control regions for RNAP III are similar in the two gene types. During oogenesis, the NOR-5S rRNA genes are amplified together with other rRNA genes and specifically transcribed in growing oocytes but not in somatic cells. In vitellogenic oocytes, the NOR-5S rRNA prevails over the canonical 5S rRNA in polysomes. This may indicate that the non-canonical ribosomes are involved in oocyte-specific translation [38].

The studies clearly showing co-localization of 5S rDNA FISH probe signals with NOR on chromosomes in some fish, such as Characidae [39], Acipenseridae [40], and Nototheniidae reviewed in [41], have encouraged us to implement a new search for the NOR-5S rRNA genes in vertebrate genomic databases.

We now present the only taxon besides turtles and crocodiles that we found to contain a 5S rRNA gene insertion in the NOR-IGSs, namely the autochthonous Antarctic ray-finned fish (Nototheniidae, Notothenioidei, Perciformes, Actinopterygii). First, we annotated the 45S ribosomal repeats, including IGS, and characterised all the putative 5S rRNA gene sequences in WGS contigs of ten species from six Nototheniidae subfamilies. We also examined the presence of different types of 5S rRNAs in the transcriptome databases available for generative and somatic organs of *Dissostichus eleginoides*, and demonstrated for the first time the existence of oocytic (maternal) and somatic 5S rRNA variants in this species.

## 2. Results

### 2.1. 5S rDNA Distribution in the Genomes of Notothens

**Figure 1:**
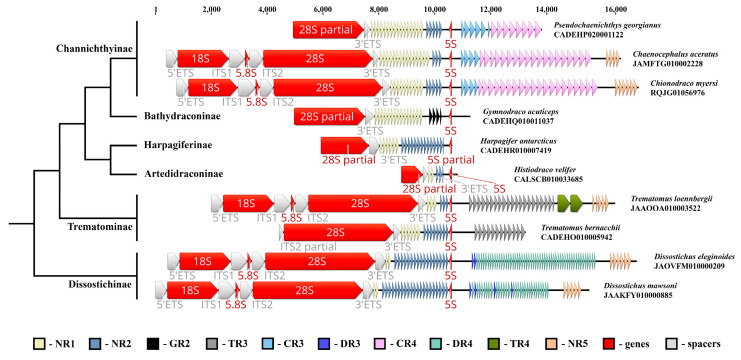
Ribosomal genes encoding the 18S, 5.8S, 28S, and 5S rRNA, found in WGS contigs of ten Nototheniidae species. NOR-5S rDNA orientation is opposite to 45S rDNA. The NOR-5S rRNA gene is located within a unique region between the IGS internal repeats. Nototheniidae phylogeny is given according to [44].

To construct a list of vertebrate genomes in which the 5S rRNA gene is incorporated in the NOR IGS sequence, we analysed all available NCBI WGS and RefSeq deuterostome databases, looking for cases where both 28S and 5S rDNA are present in the same contig. Besides turtles and crocodiles [38], the NOR associated the 5S rRNA gene was found in the IGSs of ten Nototheniidae species from six subfamilies, namely Artedidraconinae (*Histiodraco velifer*, NCBI WGS CALSCB010033685); Bathydraconinae (*Gymnodraco acuticeps*, NCBI WGS CADEHQ010011037); Channichthyinae (*Chaenocephalus aceratus*, NCBI WGS JAMFTG010002228, *Chionodraco myersi*, NCBI WGS RQJG01056976, *Pseudochaenichthys georgianus*, NCBI WGS CADEHP020001122); Harpagiferinae (*Harpagifer antarcticus*, NCBI WGS CADEHR010007419); Dissostichinae (*Dissostichus mawsoni*, NCBI WGS JAAKFY010000885, *D. eleginoides,* NCBI WGS JAOVFM010000209); and Trematominae (*Trematomus loennbergii*, NCBI WGS JAAOOA010003522, *T. bernacchii*, NCBI WGS CADEHO010005942). Complete rDNA clusters were found for *C. aceratus*, *C. myersi*, *D. mawsoni*, *D. eleginoides*, and *T. loennbergii* (Table S1). The species examined represent the main evolutionary branches of the Nototheniidae family (Figure 1) within its modern boundaries [42,43,44], which allows extrapolating the result obtained to all the representatives of the family. Thus, we can state that the 5S rRNA gene insert (NOR-5S rRNA gene) is present in the IGS in all the Nototheniidae species.

Similar to the turtle and crocodile genomes, the NOR-5S RNA gene in the IGS of Nototheniidae has an orientation opposite to the rDNA clusters and is noticeably shifted to the 28S rRNA gene (Figure 1). Nototheniidae IGSs contain tandem repeats of various types (which we called NR1-NR5, Figure S1), which is typical of other vertebrate IGS sequences. Between the 28S and NOR-5S genes, there are two family-specific tandem repeats: NR1 and NR2. At the 3′-end, the NOR-5S gene either borders on NR2 repeats (in *G. acuticeps* on genus specific GR2) or is separated from the gene itself by a short polyA sequence (Figure S2). At the 5′-end, the NOR-5S rRNA gene borders on a unique sequence, in which the first 24 nucleotides are highly conserved in all the species examined (Figure S2). This sequence differs from the spacer sequence of the canonical 5S rRNA gene cluster. Various tandem repeats (DR3, DR4, NR5 (Dissostichinae), TR3, NR5 (Trematominae), CR3, CR4, and NR5 (Channichthyinae)) are sequentially located downstream of the NOR-5S rRNA gene in the corresponding IGS sequences (Figure 1).

To compare the features of the discovered Nototheniidae NOR-5S rRNA gene with the canonical 5S rRNA genes, we searched the corresponding NCBI WGS databases for clustered 5S rRNA genes. Annotated sequences of the canonical 5S rRNA gene of the stickleback *G. aculeatus* were used as a reference. The 5S rRNA gene clusters were found in eight of the ten species examined: *H. antarcticus* (NCBI WGS CADEHR010007951), *G. acuticeps* (NCBI WGS CADEHQ010010126), *P. georgianus* (NCBI WGS CADEHK010005965), *T. bernacchii* (NCBI WGS CADEHO010006468), *D. mawsoni* (NCBI WGS JAAKFY010000822), *T. loennbergii* (NCBI WGS JAAOOA010000286), *C. myersi* (NCBI WGS RQJG01016824), and *D. eleginoides* (NCBI WGS JAOVFM010000358). In the genomic resources available for *C. aceratus* and *H. velifer*, no 5S rRNA gene clusters were found. Alignment of the clustered 5S rDNA sequences with each other shows their high degree of homology (Figure S3). The few sequence differences are probably a result of sequencing errors. We believe that the calculated consensus sequence of the 5S rRNA gene, which we will refer to as the NTS-5S rRNA gene, is the only form of the canonical 5S rRNA gene in Nototheniidae genomes.

### 2.2. 5S rRNA Types and Their Expression

**Figure 2:**
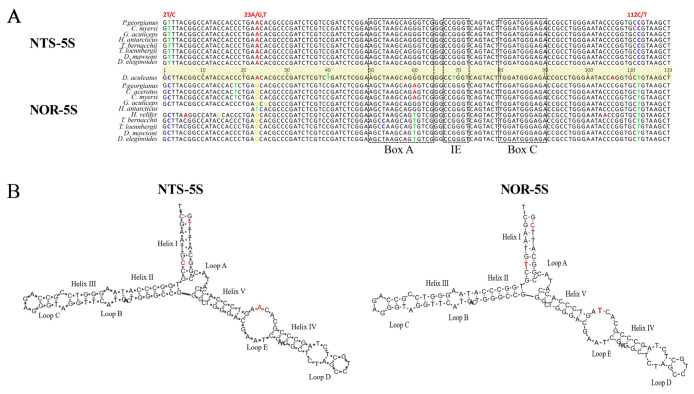
Comparison of the canonical 5S rRNA gene (NTS-5S) and the 5S rRNA gene inserted in IGS (NOR-5S) structures. (**A**) Alignment of NTS-5S and NOR-5S rRNA gene consensus sequences of Nototheniidae fish. Nucleotide substitutions at positions 2, 23, and 112 distinguishing two variants of the 5S gene are highlighted. Components of the internal control region (ICR), namely the A-box, the C-box, and the intermediate element (IE), are outlined with dotted rectangles. (**B**) Secondary structures of the Nototheniidae consensus NTS-5S and NOR-5S rRNA molecules predicted using the Andronescu’s RNA energy model at +37 °C according to [46]. The different nucleotides in the two 5S rRNA variants are marked in red.

A comparison of the NOR-5S and NTS-5S rRNA gene sequences in Nototheniidae revealed three nucleotide substitutions located at the 2nd, 23rd, and 112th positions (Figure 2A). The regulatory internal control region (ICR), known as the binding site for transcription factor TFIIIA [45], is exactly the same in the NOR-5S sequence as in the canonical 5S rRNA genes, suggesting the functionality of the former. An additional fact speaking in favour of NOR-5S functionality was obtained from the 5S rRNA secondary structure modelling. We showed that the consensus NOR-5S rRNA sequence forms a secondary structure typical of the canonical 5S rRNA molecule (Figure 2B).

**Figure 3:**
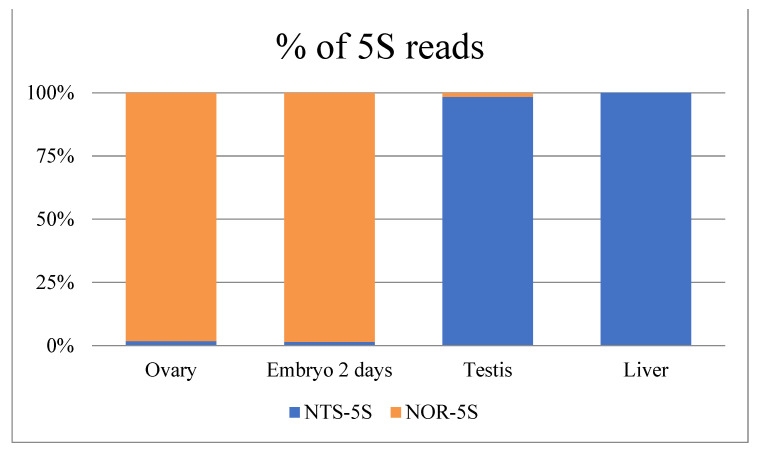
Different representation of the NOR-5S and NTS-5S rRNA sequencing reads in different tissue transcriptomes of *Dissostichus eleginoides* (Dissostichinae). Percentage of total 5S rRNA sequencing reads was calculated from NCBI BioProjects PRJNA511578 data.

This three nucleotide difference was used to discriminate between the closely related NTS-5S and NOR-5S rRNA variants in the nototheniids. We analysed the Patagonian toothfish transcriptome data of NCBI BioProjects PRJNA511578 (Figure 3) and PRJNA864592 (Figure S4) to identify the representation of NTS-5S and NOR-5S rRNAs in different tissues. It should be noted that rRNAs are removed in the course of routine transcriptomic analysis, so the expression of 5S rRNA variants may be underestimated. However, an abundance of NOR-5S transcripts was found only in the ovary and early embryo, being practically absent in the testis and adult somatic tissues (Figure 3 and Figure S4). These results are very similar to the findings obtained in turtles *Mauremys reevesii* and *Trachemys scripta*, where NOR-5S rRNAs were detected in ovary transcriptome and in oocytes by RT-PCR, respectively [38]. The predominant NOR-5S rRNA in toothfish ovaries and early embryos is considered to be maternal 5S rRNA involved in early development.

## 3. Discussion

In this work, we demonstrate the 5S rRNA gene inserted into NOR-IGS sequences in ten species of the Nototheniidae fish. Compared to the canonical NTS-5S rRNA gene, the NOR-5S rDNA insertion has three nucleotide substitutions that do not disrupt the secondary structure of the RNA transcript. Analysis of *D. eleginoides* transcriptome databases revealed NOR-5S rRNA in the ovaries and early embryos. The results indicate that the detected insertion is functional. It seems to be characteristic of the IGS of all the members of Nototheniidae, a family of a geographically limited habitat, mostly endemic to the Southern Ocean. Along with Testudines and Crocodilia in reptiles [38], this is a new reliable example of this situation in Deuterostoia. 

The detection of a functional insertion of the 5S rRNA gene into the NOR-IGS sequence in Nototheniidae fish seems to be an important result. According to a few histological studies of ovaries in toothfish, the Nototheniidae family species are characterised by formation of multiple extrachromosomal nucleoli in oocyte nuclei caused by rRNA gene amplification during oogenesis [47,48,49]. In vertebrates, the NOR amplification phenomenon in oogenesis is typical of Chondrostei and Teleostei among fish, of all amphibians, and of Testudines and Crocodilia among reptiles. Nucleolar genes, thousand-fold amplified, are transcribed into a thousand-fold increased number of 45S pre-rRNA molecules. In late oocytes, rRNA is an essential part of maternal RNA, stockpiled for early embryonic development, for a review, see [50]. These rRNA molecules are components of the maternal ribosomes that provide protein synthesis in the early embryo before embryonic ribosomes are synthesised. A recent study by Leesch and co-authors convincingly demonstrated a mechanism of maternal ribosome suppression in late oocytes prior to fertilisation [51]. In amphibian embryos, the rRNA genes are switched on shortly after the midblastula stage [52]. In oocytes with amplified nucleoli, the quantitative rRNA balance between 45S and 5S rRNA should ensure formation of the required number of ribosomes to support early embryonic translation. At present, we see two strategies that are used to maintain the balance. (1) The extra NOR clusters of specific oocyte 5S genes were found on many chromosomes in the genomes of amphibians [53] and some fish [15]. They are activated in oocytes but not in somatic tissues. However, we do not know how their activity is regulated in balance with the activity of amplified 45S rDNA repeats. (2) In Testudines, Crocodilia, and Nototheniidae, the oocyte 5S rRNA gene is inserted within the NOR-IGS sequence [38], also shown in this research. This strategy provides for a quantitatively balanced amplification of all four ribosomal genes. However, again, we know nothing about the regulation of the transcription balance between the 45S pre-rRNA and 5S rRNA genes on amplified NOR repeats.

Similar to other eukaryotic groups (yeast [54], the Asteraceae family [55], and a few invertebrate species [56,57,58,59], as well as turtles and crocodiles among vertebrates [38]), the NOR-5S rRNA gene orientation is opposite to the cluster of ribosomal genes. It should be transcribed by RNAP III in the direction opposite to the 45S pre-rRNA synthesis by RNAP I. The anti-parallel orientation of the NOR-5S gene in the IGS sequence appears to prevent its transcription by RNAP I. A small amount of NOR-5S rRNA molecules in the testis and spleen transcriptomes (Figure 3 and Figure S4) may be due to either random reading during sequencing or appearance of unexpected transcripts as a result of RNAP errors. 

As for the origin of the NOR-5S rDNA insert, one can make assumptions. We did not find any traces of mobile elements in the NOR-5S environment. However, we would not exclude the role of transposons in this insertion. In many animal taxa, rDNA loci were found to be the niche for mobile elements [60]. The strong intrafamily conservation and wide distribution of transposable elements across Nototheniidae species [61] supports this suggestion.

We found the NOR-5S rRNA genes in six nototheniid subfamilies for which WGS contigs with 5S rRNA genes were available. At present, only two phylogenetic branches among Vertebrata are known to have the NOR-5S rRNA genes in their genome. They are the Archelosauria clade of reptiles [38] and the Nototheniidae fish (this study). The difference between the NTS-5S and NOR-5SrRNA genes in turtles and crocodiles ranges from 23 (*T. scrypta*) to 26 (*Alligator mississippiensis*) nucleotide substitutions [38] compared the notothens (three nucleotide substitutions). Currently, the merged Nototheniidae family was thought to be formed about 42 million years ago (mya) in course of the Antarctic ice shield formation [42,44]. The evolutionary lineage of Archelosauria is much older, having evolved around 270 mya [62]. Thus, despite the similarities between Nototheniidae and Archelosauria, it is clear that the insertion of 5S rDNA into their IGS occurred independently.

Our findings in notothens can explain cytogenetic data obtained earlier. Two-colour hybridisation revealed colocalization of 5S and 45S rDNA on chromosomes of Nototheniidae fish (reviewed in [41], see also [63]). This chromosomal synteny of the two rRNA gene families is maintained in *D. mawsoni*, where the ribosomal genes locus is duplicated [64]. It is worth mentioning that in the sub-Antarctic clade, namely in chromosome sets of the Bovichtidae species, the 5S ribosomal genes are located far from the 45S genes on different pairs of chromosomes [65]. Together with our data, this proves that the integration of 5S and NOR rRNA genes occurred prior to Nototheniidae lineage diversification and confirms the validity of the last classifications proposed for the suborder Notothenioidei [42,43,44].

## 4. Materials and Methods

The search for nucleotide sequences containing fragments of the rRNA genes and IGS was performed using the BLAST algorithm applied to the NCBI WGS and RefSeq databases. To identify the 28S and 18S rRNA genes, we used the corresponding evolutionary conserved sequences from the chicken rRNA gene cluster [66] as a reference. To identify the 5S rRNA genes, we used annotated nucleotide sequences of *Gasterosteous aculeatus* (Gasterosteidae, Scorpaeniformes) (NCBI accession number: NC_053219.1:10986445-10986563) as a reference. We also used our data on both NOR-5S and NTS-5S rRNA genes of turtles and crocodiles [38]. Reference assembly, coverage assessment and sequence alignment and annotation, as well as 5S rRNA secondary structure modelling were performed using Geneious 9.1 (https://www.geneious.com/). NOR-5S and NTS-5S rRNA reference mapping was carried out using the Geneious mapper. The maximum number of allowable mismatches or gaps per read and per both read and reference sequence was 1%. 

To identify and differentiate NOR-5S and NTS-5S rRNA transcripts in different tissues, we analysed raw transcriptome sequencing data of Patagonian toothfish (*Dissostichus eleginoides*) available from NCBI BioProjects PRJNA511578 and PRJNA864592. *D. eleginoides* transcriptomes from the testis (NCBI SRA SRX5558183), ovary (NCBI SRA SRX5558184, SRX16793492), 2-day-old embryo (NCBI SRA SRX5558185), liver (NCBI SRA SRX5558187, SRX16793496), kidney (NCBI SRA SRX16793495), heart (NCBI SRA SRX16793493), and spleen (NCBI SRA SRX16793498) were analysed. Alignment of the transcripts to the NOR-5S rDNA sequences of *D.eleginoides* was performed using the Geneious algorithm. The 5S rRNA secondary structures were predicted according to Andronescu’s RNA energy model at +37 °C [46]. Multiple sequence alignment was performed using the Clustal W [67] and MAFFT [68] methods.

## 5. Conclusions

This study explores the colocalization of the 5S and 45S ribosomal genes, previously reported only for Testudines and Crocodilia species, in a wider number of vertebrate genomes. Most likely, the inclusion of the 5S rRNA gene in the NOR-IGS sequence contributed to the evolutionary success of the taxa, characterised by NOR amplification during oogenesis. The biological meaning of the colocalization of the 5S and 45S ribosomal genes seems to be the same in notothens, turtles, and crocodiles; it provides for a simultaneous and quantitatively coordinated amplification of all four rRNA genes. It is possible that 5S rRNA polymorphism leads to the formation of heterogeneous ribosomes and translation of different mRNAs. This would be important for the regulation of the expression at different stages of ontogenesis. Thus, there is a chance that further advances in deciphering difficult-to-assemble genomic regions, such as rDNA IGSs, will reveal more taxa with NOR-5S insertions in the genome.

## Data Availability

No new data were created in this study. The search results of publicly open data presented in this study are available in the text body or Supplementary Material.

## Supplementary Materials

The supporting information can be downloaded at: https://www.mdpi.com/article/10.3390/ijms24087376/s1.
