# Supplementary material for: Single Copies of the 5S rRNA Inserted into 45S rDNA Intergenic Spacers in the Genomes of Nototheniidae (Perciformes, Actinopterygii)
Source: Int J Mol Sci. 2023 Apr 17;24(8):7376. doi: 10.3390/ijms24087376 (PMC10138776; doi:10.3390/ijms24087376)

Single copies of the 5S rRNA gene inserted into 45S rDNA intergenic spacers in the genomes of Nototheniidae (Perciformes, Actinopterygii)

Alexander Dyomin, Svetlana Galkina, Arina Ilina, Elena Gaginskaya

Supplementary material

Table S1. rDNA elements found in genome sequences of Nototheniidae representatives

Species	NCBI whole genome shotgun sequence accession number	rDNA elements found in the genome sequence
	Artedidraconinae	
Histiodraco velifer	CALSCB010033685	rDNA repeat partial
	Bathydraconinae	
Gymnodraco acuticeps	CADEHQ010011037	rDNA repeat partial
	Channichthyinae	
Chaenocephalus aceratus	JAMFTG010002228	2 Full rDNA repeats
Chionodraco myersi	RQJG01056976	rDNA repeat partial
Pseudochaenichthys georgianus	CADEHP020001122	rDNA repeat partial
	Harpagiferinae	
Harpagifer antarcticus	CADEHR010007419	rDNA repeat partial
	Dissostichinae	
Dissostichus mawsoni	JAAKFY010000885	1 full rDNA repeats
Dissostichus eleginoides	JAOVFM010000209	1 full rDNA repeats, 2 IGS
	Trematominae	
Trematomus loennbergii	JAAOOA010003522	1 full rDNA repeats, 1 partial rDNA repeats
Trematomus bernacchii	CADEHO010005942	rDNA repeat partial

Figure S1. IGS repeats in Nototheniidae. The alignment of consensus repeat sequences found for each species is presented.

Repeat NR1 found in Trematominae (*Trematomus loennbergii*, *T. bernacchii*), Dissostichinae (*Dissostichus mawsoni*, *D. eleginoides*), Channichthyinae (*Chionodraco myersi*, *Chaenocephalus aceratus*, *Pseudochaenichthys georgianus*), Bathydraconinae (*Gymnodraco acuticeps*), Harpagiferinae (*Harpagifer antarcticus*), Artedraconinae (*Histiodraco velifer)*

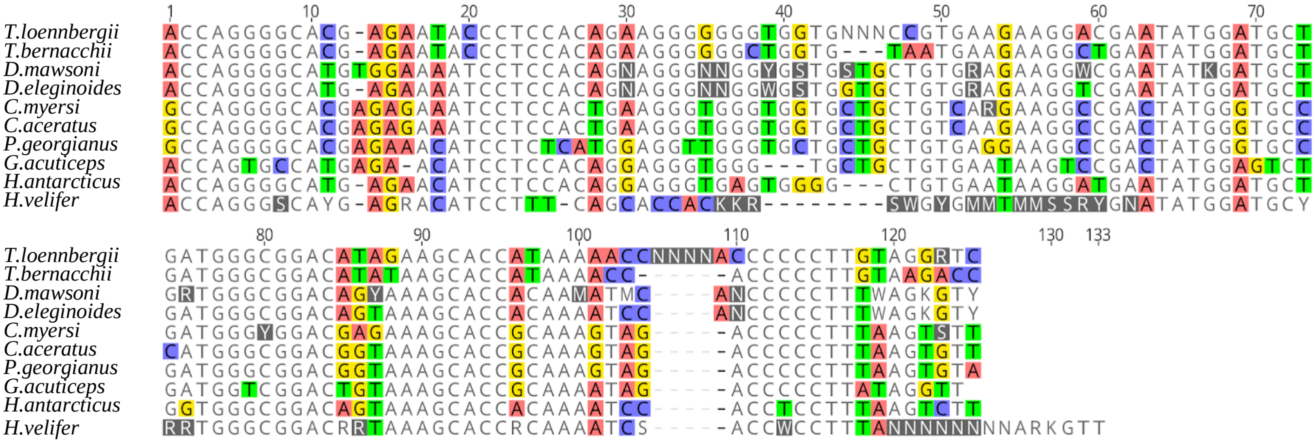

Repeat NR2 found in Trematominae (*Trematomus loennbergii*, *T. bernacchii*), Dissostichinae (*Dissostichus mawsoni*, *D. eleginoides*), Channichthyinae (*Chaenocephalus aceratus*, *Chionodraco myersi*, *Pseudochaenichthys georgianus*), Harpagiferinae (*Harpagifer antarcticus*), Artedraconinae (*Histiodraco velifer)*

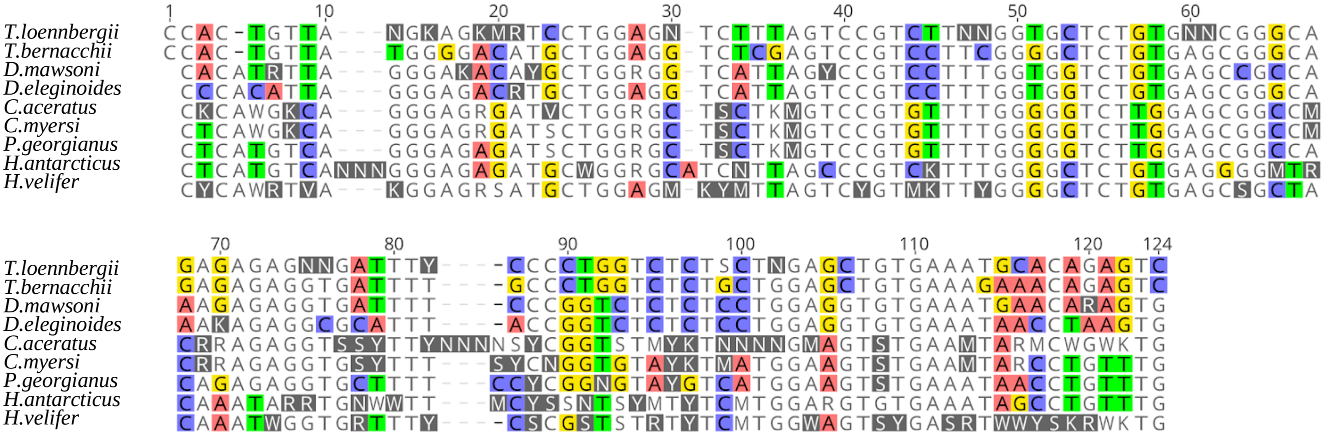

Repeat GR2 found in Bathydraconinae (*Gymnodraco acuticeps*)

G.acuticeps ATTATGAGGCACTATTTCGGGATCCATTTTCACCATTTTAATCCCTTTCTCTGGTTCTTCCAAMAGT
TAACTTRTACTTATTTAGCCTGTGGAKGAATGCAAGGGTAGTTATCAGGGGACTCTACCTGC

Repeat DR3 found in Dissostichinae (*Dissostichus mawsoni*, *D. eleginoides*)

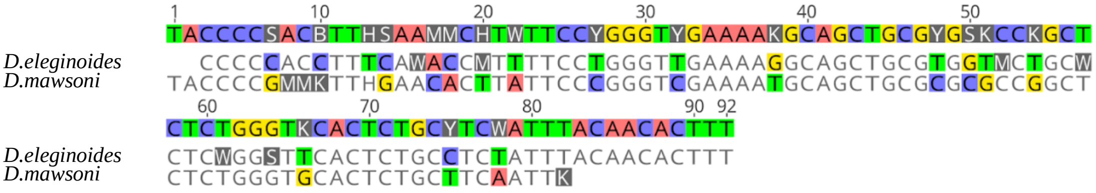

Repeat TR3 found in Trematominae (*Trematomus loennbergii*, *T. bernacchii*)

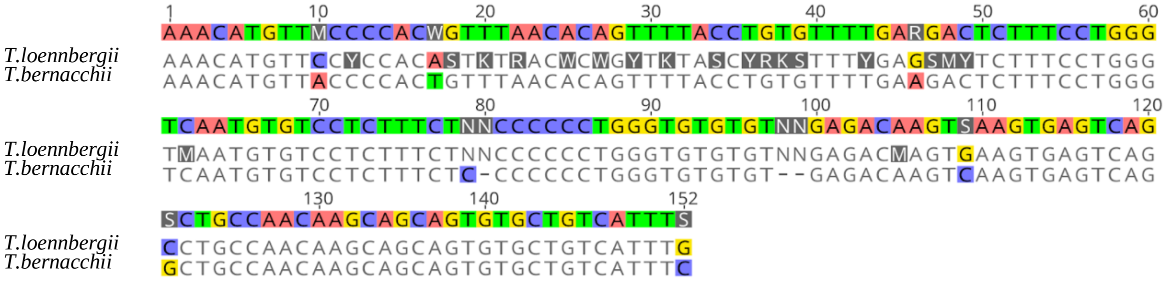

Repeat CR3 found in Channichthyinae (*Chionodraco myersi*, *Chaenocephalus aceratus*, *Pseudochaenichthys georgianus*)

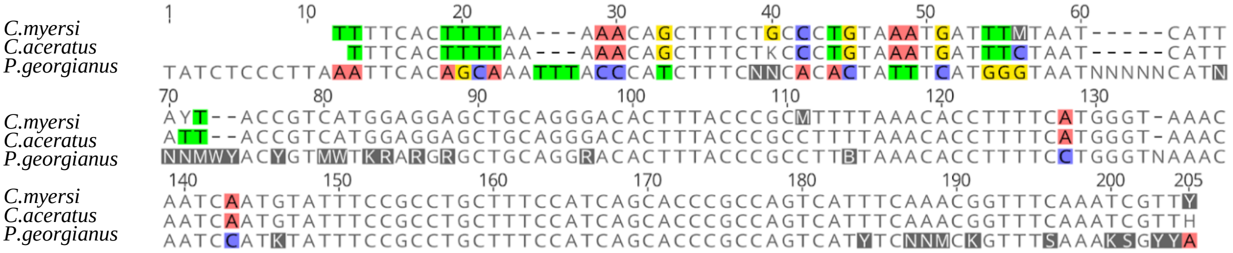

Repeat DR4 found in Dissostichinae (*Dissostichus mawsoni*, *D. eleginoides*)

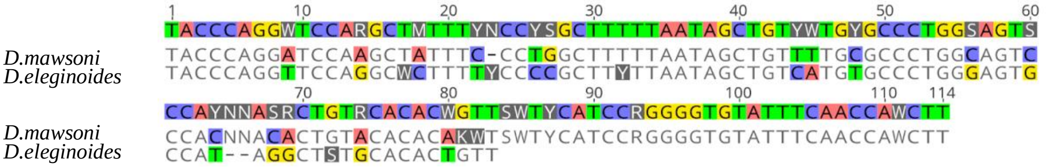

Repeat CR4 found in Channichthyinae (*Chionodraco myersi*, *Chaenocephalus aceratus*, *Pseudochaenichthys georgianus*)

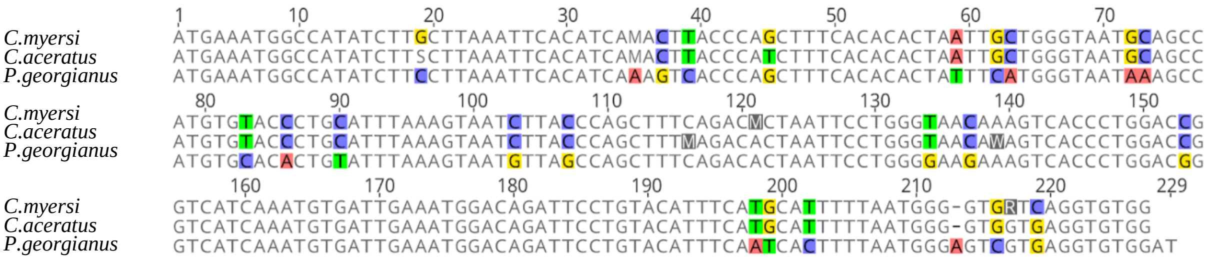

Repeat NR5 found in Dissostichinae (*Dissostichus mawsoni*, *D. eleginoides*), Trematominae (*Trematomus loennbergii*), Channichthyinae (*Chaenocephalus aceratus*, *Chionodraco myersi*)

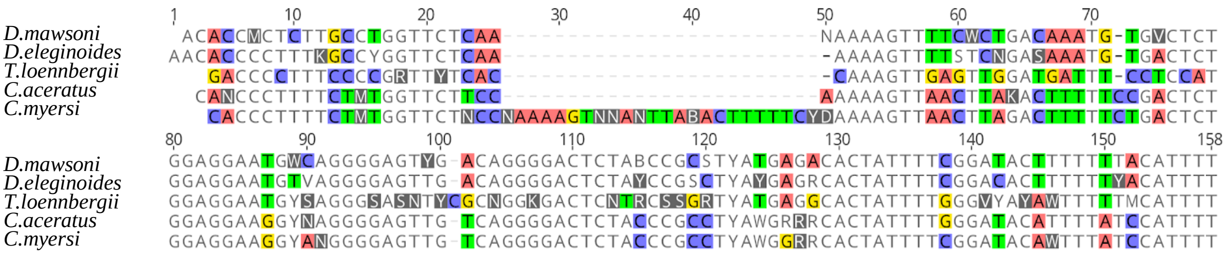

Figure S2. NOR-5S gene environment in IGS of Nototheniidae fishes. (A) Alignment of ~240 bp flanking sequences at the 3'-end of the NOR-5S gene shows short polyA sequence (highlighted in yellow). (B) Alignment of ~850 bp flanking sequences at the 5'-end of the NOR-5S gene reveals a conserved motif of 24 nucleotides (highlighted in yellow).

A. Conserved unique sequence at the 3'- end of the NOR-5S gene

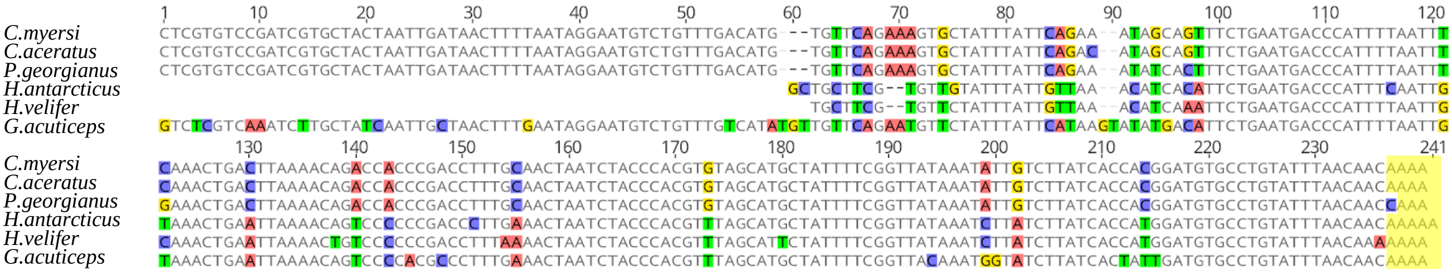

B. Conserved unique sequence at the 5'- end of the NOR-5S gene

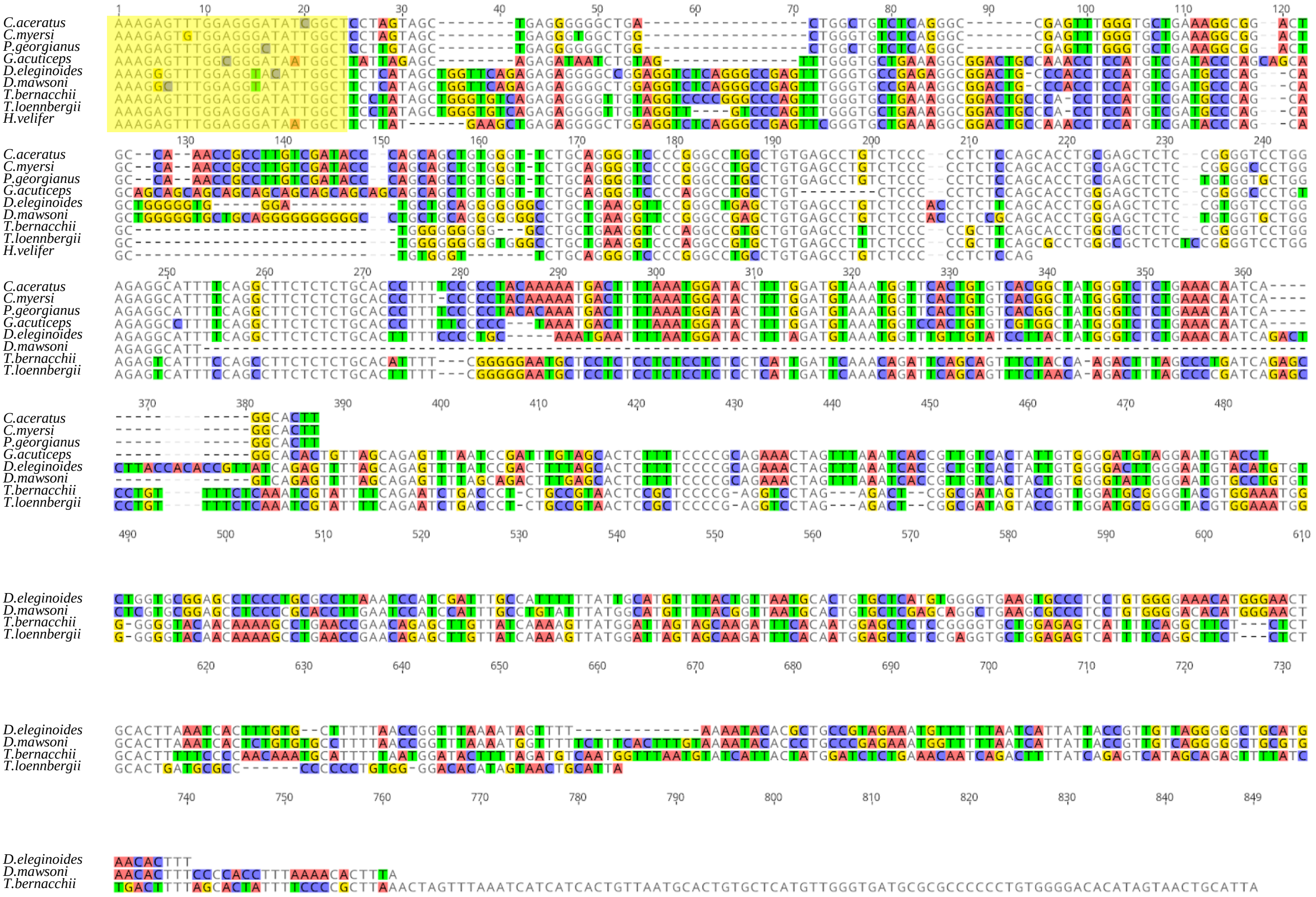

Figure S3. NTS-5S rRNA genes mapped in WGS contigs of eight Nototheniidae species. The alignment of all NTS-5S rRNA gene copies is presented.

Pseudochaenichthys georgianus NCBI WGS CADEHK010005965

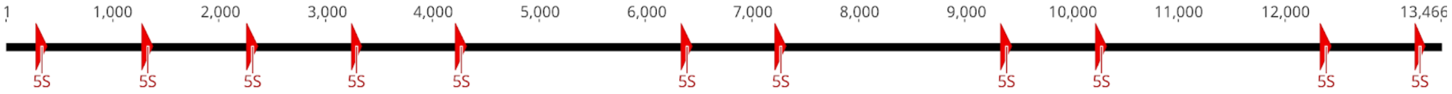


```
    1        10        20        30        40        50        60        70        80        90        100       110      119
1   GTTTACGGCCATACCACCCTGAACACGCCCGATCTCGTCCGATCTCGGAAGCTAAGCAGGGTCGGGCCGGGTCAGTACTTGGATGGGAGACCGCCTGGGAATACCCGGTGCCGTAAGCT
2   GTTTACGGCCATACCACCCTGAACACGCCCGATCTCGTCCGATCTCGGAAGCTAAGCAGGGTCGGGCCGGGTCAGTACTTGGATGGGAGACCGCCTGGGAATACCCGGTGCCGTAAGCT
3   GTTTACGGCCATACCACCCTGAACACGCCCGATCTCGTCCGATCTCGGAAGCTAAGCAGGGTCGGGCCGGGTCAGTACTTGGATGGGAGACCGCCTGGGAATACCCGGTGCCGTAAGCT
4   GTTTACGGCCATACCACCCTGAACACGCCCGATCTCGTCCGATCTCGGAAGCTAAGCAGGGTCGGGCCGGGTCAGTACTTGGATGGGAGACCGCCTGGGAATACCCGGTGCCGTAAGCT
5   GTTTACGGCCATACCACCCTGAACACGCCCGATCTCGTCCGATCTCGGAAGCTAAGCAGGGTCGGGCCGGGTCAGTACTTGGATGGGAGACCGCCTGGGAATACCCGGTGCCGTAAGCT
6   GTTTACGGCCATACCACCCTGAACACGCCCGATCTCGTCCGATCTCGGAAGCTAAGCAGGGTCGGGCCGGGTCAGTACTTGGATGGGAGACCGCCTGGGAATACCCGGTGCCGTAAGCT
7   GTTTACGGCCATACCACCCTGAACACGCCCGATCTCGTCCGATCTCGGAAGCTAAGCAGGGTCGGGCCGGGTCAGTACTTGGATGGGAGACCGCCTGGGAATACCCGGTGCCGTAAGCT
8   GTTTACGGCCATACCACCCTGAACACGCCCGATCTCGTCCGATCTCGGAAGCTAAGCAGGGTCGGGCCGGGTCAGTACTTGGATGGGAGACCGCCTGGGAATACCCGGTGCCGTAAGCT
9   GTTTACGGCCATACCACCCTGAACACGCCCGATCTCGTCCGATCTCGGAAGCTAAGCAGGGTCGGGCCGGGTCAGTACTTGGATGGGAGACCGCCTGGGAATACCCGGTGCCGTAAGCT
10  GTTTACGGCCATACCACCCTGAACACGCCCGATCTCGTCCGATCTCGGAAGCTAAGCAGGGTCGGGCCGGGTCAGTACTTGGATGGGAGACCGCCTGGGAATACCCGGTGCCGTAAGCT
11  GTTTACGGCCATACCACCCTGAACACGCCCGATCTCGTCCGATCTCGGAAGCTAAGCAGGGTCGGGCCGGGTCAGTACTTGGATGGGAGACCGCCTGGGAATACCCGGTGCCGTAAGCT
```

Chionodraco myersi NCBI WGS RQJG01016824

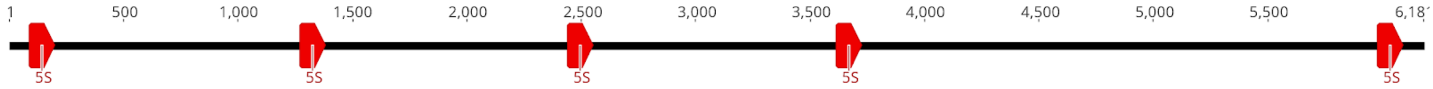


```
     1        10        20        30        40        50        60        70        80        90        100       110      119
1.1  GTTTACGGCCATACCACCCTGAACACGCCCGATCTCGTCCGATCTCGGAAGCTAAGCAGGGTCGGGCCGGGTCAGTACTTGGATGGGAGACCGCCTGGGAATACCCGGTGCCGTAAGCT
2.2  GTTTACGGCCATACCACCCTGAACACGCCCGATCTCGTCCGATCTCGGAAGCTAAGCAGGGTCGGGCCGGGTCAGTACTTGGATGGGAGACCGCCTGGGAATACCCGGTGCCGTAAGCT
3.3  GTTTACGGCCATACCACCCTGAACACGCCCGATCTCGTCCGATCTCGGAAGCTAAGCAGGGTCGGGCCGGGTCAGTACTTGGATGGGAGACCGCCTGGGAATACCCGGTGCCGTAAGCT
4.4  GTTTACGGCCATACCACCCTGAACACGCCCGATCTCGTCCAATCTCGGAAGCTAAGCAGGGTCGGGCCGGGTCAGTACTTGGATGGGAGACCGCCTGGGAATACCCGGTGCCGTAAGCT
5.5  GTTTACGGCCATACCACCCTGAACACGCCCGATCTCGTCCGATCTCGGAAGCTAAGCAGGGGCGGGCCGGGTCAGTACTTGGATGGGAGACCGCCTGGGAATACCCGGTGCCGTAAGCT
```

Gymnodraco acuticeps NCBI WGS CADEHQ010010126

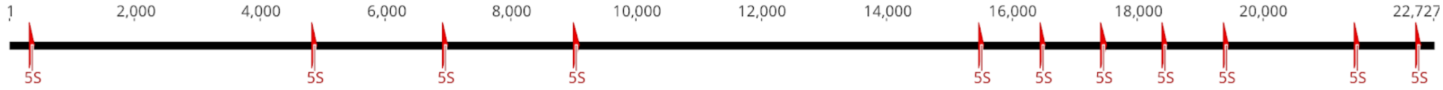


```
    1        10        20        30        40        50        60        70        80        90        100       110      119
1   GTTTACGGCCATACCACCCTGAACACGCCCGATCTCGTCCGATCTCGGAAGCTAAGCAGGGTCGGGCCGGGTCAGTACTTGGATGGGAGACCGCCTGGGAATACCCGGTGCCGTAAGCT
2   GTTTACGGCCATACCACCCTGAACACGCCCGATCTCGTCCGATCTCGGAAGCTAAGCAGGGTCGGGCCGGGTCAGTACTTGGATGGGAGACCGCCTGGGAATACCCGGTGCCGTAAGCT
3   GTTTACGGCCATACCACCCTGAACACGCCCGATCTCGTCCGATCTCGGAAGCTAAGCAGGGTCGGGCCGGGTCAGTACTTGGATGGGAGACCGCCTGGGAATACCCGGTGCCGTAAGCT
4   GTTTACGGCCATACCACCCTGAACACGCCCGATCTCGTCCGATCTCGGAAGCTAAGCAGGGTCGGGCCGGGTCAGTACTTGGATGGGAGACCGCCTGGGAATACCCGGTGCCGTAAGCT
5   GTTTACGGCCATACCACCCTGAACACGCCCGATCTCGTCCGATCTCGGAAGCTAAGCAGGGTCGGGCCGGGTCAGTACTTGGATGGGAGACCGCCTGGGAATACCCGGTGCCGTAAGCT
6   GTTTACGGCCATACCACCCTGAACACGCCCGATCTCGTCCGATCTCGGAAGCTAAGCAGGGTCGGGCCGGGTCAGTACTTGGATGGGAGACCGCCTGGGAATACCCGGTGCCGTAAGCT
7   GTTTACGGCCATACCACCCTGAACACGCCCGATCTCGTCCGATCTCGGAAGCTAAGCAGGGTCGGGCCGGGTCAGTACTTGGATGGGAGACCGCCTGGGAATACCCGGTGCCGTAAGCT
8   GTTTACGGCCATACCACCCTGAACACGCCCGATCTCGTCCGATCTCGGAAGCTAAGCAGGGTCGGGCCGGGTCAGTACTTGGATGGGAGACCGCCTGGGAATACCCGGTGCCGTAAGCT
9   GTTTACGGCCATACCACCCTGAACACGCCCGATCTCGTCCGATCTCGGAAGCTAAGCAGGGTCGGGCCGGGTCAGTACTTGGATGGGAGACCGCCTGGGAATACCCGGTGCCGTAAGCT
10  GTTTACGGCCATACCACCCTGAACACGCCCGATCTCGTCCGATCTCGGAAGCTAAGCAGGGTCGGGCCGGGTCAGTACTTGGATGGGAGACCGCCTGGGAATACCCGGTGCCGTAAGCT
11  GTTTACGGCCATACCACCCTGAACACGCCCGATCTCGTCCGATCTCGGAAGCTAAGCAGGGTCGGGCCGGGTCAGTACTTGGATGGGAGACCGCCTGGGAATACCCGGTGCCGTAAGCT
```

Harpagifer antarcticus CADEHR010007951

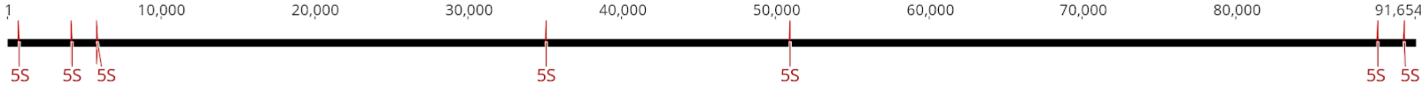


```
     1        10        20        30        40        50        60        70        80        90        100       110      119
1.1  GTTTACGGCCATACCACCCTGAACACGCCCGATCTCGTCCGATCTCGGAAGCTAAGCAGGGTCGGGCCGGGTCAGTACTTGGATGGGAGACCGCCTGGGAATACCCGGTGCCGTAAGCT
2.2  GTTTACGGCCATACCACCCTGAACACGCCCGATCTCGTCCGATCTCGGAAGCTAAGCAGGGTCGGGCCGGGTCAGTACTTGGATGGGAGACCGCCTGGGAATACCCGGTGCCGTAAGCT
3.3  GTTTACGGCCATACCACCCTGAACACGCCCGATCTCGTCCGATCTCGGAAGCTAAGCAGGGTCGGGCCGGGTCAGTACTTGGATGGGAGACCGCCTGGGAATACCCGGTGCCGTAAGCT
4.4  GTTTACGGCCATACCACCCTGAACACGCCCGATCTCGTCCGATCTCGGAAGCTAAGCAGGGTCGGGCCGGGTCAGTACTTGGATGGGAGACCGCCTGGGAATACCCGGTGCCGTAAGCT
5.5  GTTTACGGCCATACCACCCTGAACACGCCCGATCTCGTCCGATCTCGGAAGCTAAGCAGGGTCGGGCCGGGTCAGTACTTGGATGGGAGACCGCCTGGGAATACCCGGTGCCGTAAGCT
6.6  GTTTACGGCCATACCACCCTGAACACGCCCGATCTCGTCCGATCTCGGAAGCTAAGCAGGGTCGGGCCGGGTCAGTACTTGGATGGGAGACCGCCTGGGAATACCCGGTGCCGTAAGCT
7.7  GTTTACGGCCATACCACCCTGAACACGCCCGATCTCGTCCGATCTCGGAAGCTAAGCAGGGTCGGGCCGGGTCAGTACTTGGATGGGAGACCGCCTGGGAATACCCGGTGCCGTAAGCT
```

Trematomus loennbergii NCBI WGS JAAOOA010000286

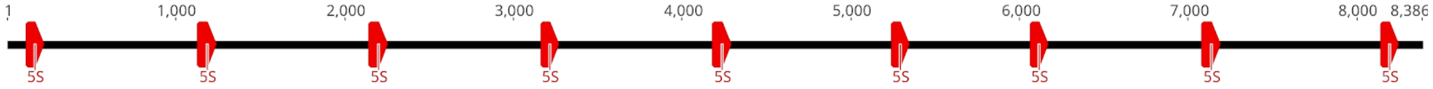


```
     1        10        20        30        40        50        60        70        80        90        100       110      119
1.1  GTTTACGGCCATACCACCCTGAACACGCCCGATCTCGTCCGATCTCGGAAGCTAAGCAGGGTCGGGCCGGGTCAGTACTTGGATGGGAGACCGCCTGGGAATACCCGGTGCCGTAAGCT
2.2  GTTTACGGCCATACCACCCTGAACACGCCCGATCTCGTCCGATCTCGGAAGCTAAGCAGGGTCGGGCCGGGTCAGTACTTGGATGGGAGACCGCCTGGGAATACCCGGTGCCGTAAGCT
3.3  GTTTACGGCCATACCACCCTGAACACGCCCGATCTCGTCCGATCTCGGAAGCTAAGCAGGGTCGGGCCGGGTCAGTACTTGGATGGGAGACCGCCTGGGAATACCCGGTGCCGTAAGCT
4.4  GTTTACGGCCATACCACCCTGAACACGCCCGATCTCGTCCGATCTCGGAAGCTAAGCAGGGTCGGGCCGGGTCAGTACTTGGATGGGAGACCGCCTGGGAATACCCGGTGCCGTAAGCT
5.5  GTTTACGGCCATACCACCCTGAACACGCCCGATCTCGTCCGATCTCGGAAGCTAAGCAGGGTCGGGCCGGGTCAGTACTTGGATGGGAGACCGCCTGGGAATACCCGGTGCCGTAAGCT
6.6  GTTTACGGCCATACCACCCTGAACACGCCCGATCTCGTCCGATCTCGGAAGCTAAGCAGGGTCGGGCCGGGTCAGTACTTGGAT-GGAGACCGCCTGGGAATACCCGGTGCCGTAAGCT
7.7  GTTTACGGCCATACCACCCTGAACACGCCCGATCTCGTCCGATCTCGGAAGCTAAGCAGGGTCGGGCC-GGTCAGTACTTGGATGGGAGACCGCCTGGGAATACCCGGTGCCGTAAGCT
8.8  GTTTACGGCCATACCA-CCTGAACACGCCCGATCTCGTCCGATCTCGGAAGCTAAGCAGGGTCGGGCCGGGTCAGTACTTGGATGGGAGACCGCCTGGGAATACCCGGTGCCGTAAGCT
9.9  GTTTACGGCCATACCACCCTGAACACGCCCGATCTCGTCCGATCTCGGAAGCTAAGCAGGGTCGGGCCGGGTCAGTACTTGGAG-GGAGACCGCCTGGGAATACCC-GTGCCGTAAGCT
```

Trematomus bernacchii NCBI WGS CADEHO010006468

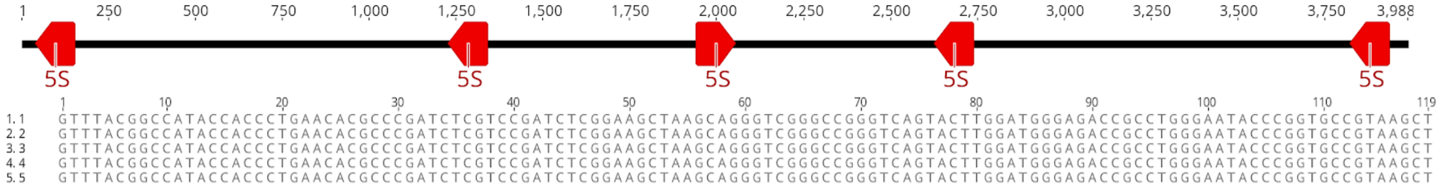

Dissostichus eleginoides NCBI WGS JAOVFM010000358

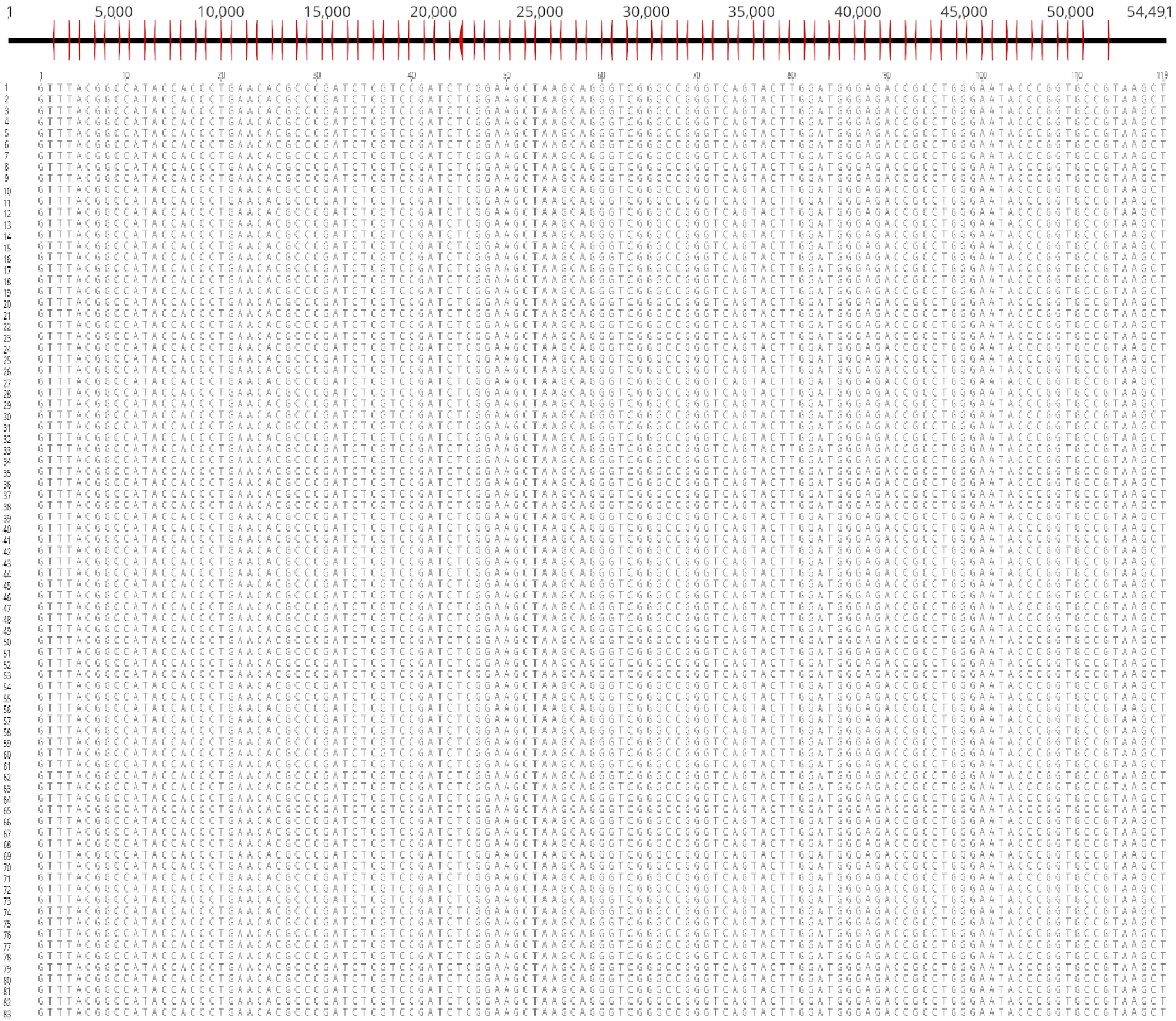

Dissostichus mawsoni NCBI WGS JAAKFY010000822

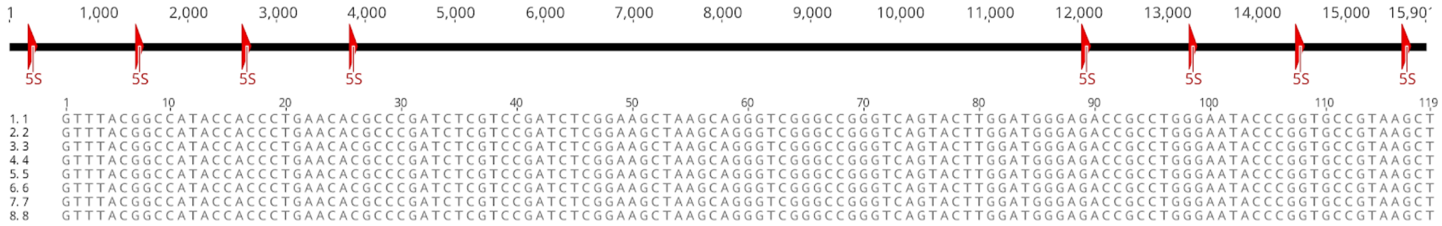

Figure S4. Different representation of the NOR-5S and NTS-5S rRNA sequencing reads in different tissue transcriptomes of *Dissostichus eleginoides* (Dissostichinae). Percentage of total 5S rRNA sequencing reads was calculated from NCBI BioProjects PRJNA864592 data.

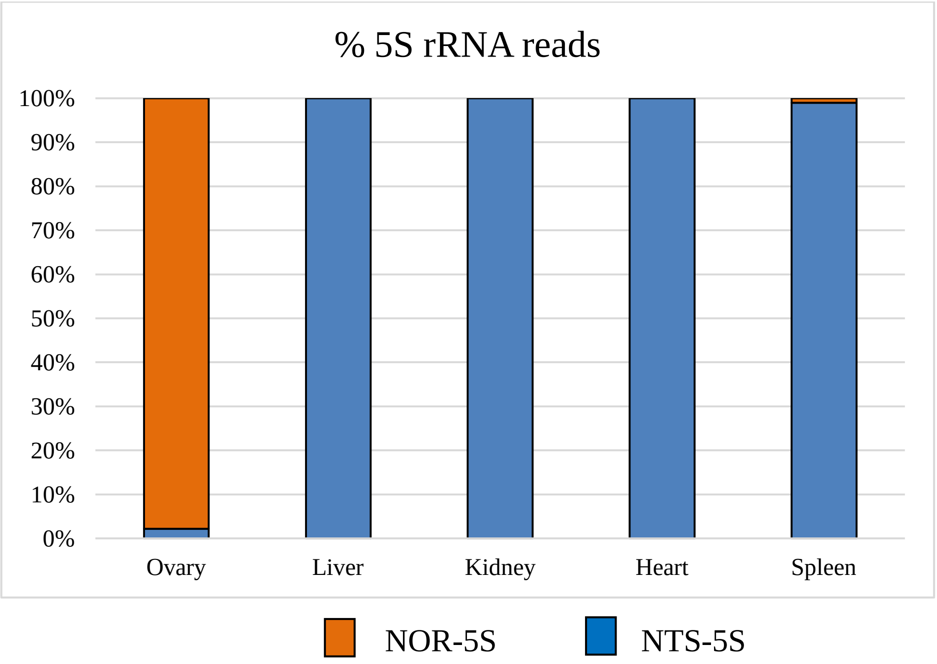

Supplement: Supplementary file 1 [file ijms-24-07376-s001.zip › ijms-2344196-supplementary.pdf]
